# Replacement value of cassava for maize in broiler chicken diets supplemented with enzymes

**DOI:** 10.5713/ajas.19.0263

**Published:** 2019-08-03

**Authors:** Edwin Peter Chang’a, Medani Eldow Abdallh, Emmanuel Uchenna Ahiwe, Said Mbaga, Ze Yuan Zhu, Fidelis Fru-Nji, Paul A de Iji

**Affiliations:** 1 Department of Animal Science, School of Environmental and Rural Science, University of New England, Armidale, New South Wales 2351, Australia; 2 Tanzania Livestock Research Institute, P.O. Box 6191, TALIRI-Uyole, Mbeya, Tanzania; 3 Department of Poultry Production, University of Khartoum, 13314, Khartoum, Sudan; 4 Department of Animal Science and Technology, Federal University of Technology, Owerri, Imo State, 1526, Nigeria; 5 Department of Animal, Aquaculture and Range Sciences, Sokoine University of Agriculture, P.O. Box 3004, Morogoro, Tanzania; 6 DSM Nutritional Products, Animal Nutrition and Health, 117440, Singapore; 7 College of Agriculture, Fisheries & Forestry, Fiji National University, P.O. Box 1544, Fiji Islands

**Keywords:** Bone Quality, Cassava, Energy Retention, Maize, Meat Yield, Performance

## Abstract

**Objective:**

Pellet durability, particle size distribution, growth response, tibia bone characteristics and energy retention were measured to evaluate cassava as an alternative energy source to replace maize in broiler diets with or without Ronozyme (A+VP) enzyme composites.

**Methods:**

A total of 480 one-day broiler chicks were randomly assigned to 8 treatments in a 4×2 factorial arrangement. Four levels of cassava: (0%, 25%, 50%, 75%) and 2 levels of enzymes (0 and 500 g/tonne) were used. Each treatment was replicated six times, with ten birds per replicate.

**Results:**

The particle size distribution in the diets showed an increasing trend of small particles with increase in cassava level. Pellet durability decreased (p<0.05) with cassava inclusion. Feed intake was highest in birds fed diets with medium cassava level at 1 to 24 d and 1 to 35 d of age. The body weight gain of birds reduced (p<0.037) as cassava level increased, but it increased (p<0.017 when enzymes were added. The feed conversion ratio was high (p<0.05) when cassava level was increased, but it reduced (p<0.05) when enzymes were added. The dressing percentage (DP), and weight of drumsticks reduced (p<0.05) with increasing cassava level. Enzyme supplementation increased (p<0.05) DP, and weight of breast, thighs and drumsticks. Ash content, weight, length, width, and bone strength decreased (p<0.05) when cassava level was increased, however, they were increased with enzyme addition. The contents of Ca, K, and Zn were raised (p<0.001) with increasing cassava level. Enzyme inclusion increased (p<0.001) all mineral contents in tibia bones. Body fat and energy retained as fat decreased (p<0.001) as cassava level increased. Enzyme inclusion increased (p<0.05) body protein content and energy retained as protein.

**Conclusion:**

Although broiler performance was depressed by high levels of cassava inclusion, it was not affected by low levels, which further improved by enzyme supplementation.

## INTRODUCTION

Broiler chickens, like other non-ruminant animals, rely on highly digestible feedstuffs, such as maize and soybean meal, as energy and protein sources, respectively [1,2]. Most conventional poultry feed ingredients, particularly maize in the tropical countries, are expensive as they are also in demand for human consumption, and may be used for bioethanol production [3,4]. Maize is the most commonly used energy source for poultry feeding worldwide [5] because it has a higher metabolizable energy (ME) content than other cereal grains [6]. In Tanzania, maize is used for feeding poultry, but is also a staple food for humans [1]. Maize production in Tanzania, as in other parts of Africa, is affected by low yield, which is attributed to drought [7], and thus it is a scarce and expensive commodity, particularly for small-to-medium (SM) scale poultry farmers. The limited supply and high cost of maize have been a threat to the SM scale poultry farmers in the country [1], thus affecting sustainability of the poultry sub-sector. Due to high and unpredictable prices of maize, use of alternative feedstuffs could be the best strategy in formulating feeds for commercial poultry production. Since Tanzania and other countries in the region are endowed with various energy-rich crops, such as sorghum, millet, sweet potatoes and cassava, these ingredients can be used to as alternative sources with a high energy content and low price for poultry feeds.

Several studies have suggested the use of cassava as a replacement for maize meal as an energy source in poultry diets because it is produced in great quantities and has high energy content [3,8,9]. Cassava can be grown without fertilizer in very arid, infertile or acidic soils that are unsuitable for maize and other crops [9]. It was reported by Augustine et al [10] that by replacing maize meal with 50% cassava meal the carcass quality of broiler chickens was improved, showing that cassava can be a reliable source of energy to substitute for maize in poultry feed. Cassava tubers have high ME; however, their inclusion in poultry diet is restricted because of their low protein content, the unbalanced amino acid profile, the high fibre content and the presence of anti-nutrients, particularly cyanogenic glucosides [4]. The anti-nutritional factors (ANF) in cassava can be reduced through several physical processing methods, including soaking, sun-drying, boiling and ensiling [11]. Microbial enzyme supplementation has also been reported to decrease the ANF of cassava [3]. Processing cassava reduces feed wastage, improves its nutritional value and maximizes poultry production [11]. A review by Omede et al [11] suggested that well-processed cassava can potentially be used to replace up to 50% of maize in poultry diets without causing adverse effects on health and performance of the birds. The use of cassava in feeds for broiler chickens could reduce the dependence on maize as a major energy source and could reduce feed costs for producers in Tanzania.

The objective of the current study was to evaluate cassava as a potential alternative source of energy to replace maize in broiler chicken diets with or without microbial enzymes. This was done by measuring the effect of different levels of cassava on its own, and with a cocktail of two enzyme products on feed quality (pellet durability and particle size distribution of diets), broiler performance, bone quality and energy retention.

## MATERIALS AND METHODS

### Dietary treatment and bird management

Four basal diets were formulated using ingredients available in Australia, which are commonly used in Tanzania, namely, maize, soybean, cottonseed and fish meals. Diets were based on the nutritional specifications for Ross 308 broiler chicks [12]. Dietary treatments contained different levels of cassava- none (0%), low (25%), medium (50%), and high (75%) replacing maize as an energy source. Each diet was fed as such or supplemented with enzymes (Ronozyme A + Ronozyme VP at 500 g/tonne of diet). Ronozyme A is an α-amylase and β-glucanase complex, while Ronozyme VP contains gluanase, cellulase, hemicellulose, and pectinase activities. Table 1 and 2 show the feed ingredients (g/kg) and nutrient composition (g/kg), respectively, of the dietary treatments, while Table 3 shows the analysed nutrient composition of tested cassava.

A total of 480 d-old Ross 308 broiler chicks with initial average weight of 38.4±0.7 g, from a local hatchery (Baiada Poultry Pty. Ltd, Tamworth, NSW, Australia), were randomly assigned to eight dietary treatments in six replicates, with 10 birds per replicate. Birds were raised in 48 deep litter floor pens (47×80×45 cm) in an environmentally controlled house at the Centre for Animal Research and Teaching, University of New England, Armidale, Australia. The initial brooding temperature was 33°C, and this was gradually reduced to 24°C±1°C at d18. Except for the first day when 24 hours of light was provided, 18 hours of lighting and 6 hours of darkness in every 24-hour period was maintained throughout the trial period. Access to feed and water was provided *ad libitum* over the trial period. A three-phase feeding programme was used with starter diets fed to birds from hatch to 10 days, grower diets from 11 to 24 days, and finisher diets from 25 to 35 days of age.

### Feed particle size distribution and pellet durability

Particle size distribution was determined by a mechanical shaker (Retsch AS 200 digit of cA, Retsch GmbH, Haan, Germany) operated at an amplitude of 3.0 mm for the duration of 5 min. Samples were sieved using sieving pans with mesh diameter of 4.40, 2.8, 2.0, 1.6, 1.25, 1 mm, and 500 μm (Retsch GmbH, Haan, Germany). Particles were classified as large (>2.8 mm), medium (1.0 to 2.8 mm) or small (<1.0 mm), and mean particle size was calculated as the discrete mean particle size (dMean) [13] following the methods described by Herrera et al [14].

Feeds were pelleted using a cold pelleter at a temperature of 60°C with 15% moisture content. The pellet durability was measured using a Seedburo pellet durability tester (Seedburo Equipment Company, Chicago, IL, USA). Approximately 500 g of pelleted grower diet samples from each treatment was screened, tumbled for 10 min and re-screened to weigh the unbroken pellets. The pellet durability index (PDI) was calculated by dividing the weight of the remaining pellets by the original weight (500 g) and multiplying by 100.

Feed leftovers and live birds were weighed and recorded to calculate the body weight gain (BWG) and feed intake (FI) at 10, 24, and 35 d of age. The feed conversion ratio (FCR) was calculated by dividing the FI values by the weight gain. Mortality in each pen (replicate) was recorded as it occurred, corrections were made accordingly. At 35 d of age, two birds from each pen were selected, weighed, electrically stunned and killed by cervical dislocation. The birds were eviscerated, and the head, neck and feet were cut off to obtain a dressed weight. Breast (without bone), thighs and drumsticks (with bones) were detached from the body, weighed and recorded to get the absolute weight of body parts which was then used to calculate the relative weight of parts meat yield as g/kg.

### Bone morphology and mineral composition

On 35 d, the left drumstick from two birds was taken from each replicate and frozen at −20°C overnight. After thawing, all the tissues adherent to the tibia bones were removed. The bones (including fat in the bone marrow) were then weighed, and length and width were measured using digital callipers. Tibia bone length was measured from the proximal end to the distal end and the width at the medial diaphysis. The breaking strength of the tibia bone was measured by positioning a 10 mm diameter compression rod against the bones and applying pressure (Lloyd, Hampshire, UK). Breaking strength was recorded as the force required to break the bone and was measured in the range of 0 to 500 N. The entire bones were then dried for 12 h at 105°C in a forced-air convection oven (Qualtex Universal Series 2000, Watson Victor Ltd, Perth, Australia) and ashed (550°C for 4 h) in a Carbolite CWF 1200 chamber furnace (Carbolite, Sheffield, UK). The ashed bone samples were ground and stored at 4°C in an airtight plastic container for dry matter and mineral content analyses.

### Whole carcass processing and energy retention

Twelve chicks were electrically stunned and killed using cervical dislocation method on day one to obtain the baseline data of body composition. On day 24, two other birds per pen were killed and immediately freeze-stored (–20°C) as whole intact bodies. The stored samples were chopped and minced together as a replicate. Two sub-samples of around 250 g each were taken from the mince, lyophilized and ground. Moisture content was calculated automatically by subtracting dry sample weight from wet weight. Ground dry samples were then analysed for crude protein (CP), gross energy, and ether extract.

Energy retention was calculated according to Olukosi et al [15]. Thereafter, energy retained as fat (RE_fat_) and as protein (RE_protein_) was calculated using the following formulae:

$$\begin{array}{l} {\text{RE}}_{\text{fat}}\;(\text{kJ})=\text{Carcass fat }(\text{g})\times 38.2\;\text{kJ}/\text{g}\\ {\text{RE}}_{\text{protein}}\;(\text{kJ})=\text{Carcass CP content }(\text{g})\times 23.6\;\text{kJ}/\text{g} \end{array}$$

The constants 38.2 and 23.6 kJ/g are the energy values per gram of fat and protein, respectively, as derived by Larbier and Leclercq [16].

Carcass CP and fat content at 24 d were computed as:

$$\begin{array}{l} \text{Carcass CP content}=\text{CP }\%\times \text{live body weight}\\ \text{Body fat content}=\text{Fat }\%\times \text{live body weight} \end{array}$$

### Statistical analyses

Complete randomization was applied in this study, and data on FI, BWG, FCR, meat parts yield and tibia bone morphology and mineral composition were analysed in a two-way analysis of variance using the general linear model procedure of Minitab statistical software, version 17 [17]. Tukey’s pairwise comparison test was used to separate the differences between mean values at the p≤0.05 level of probability.

### Animal ethics

This experiment was approved by the Animal Ethics Committee of the University of New England, with approval number AEC17-079. All management and procedures in this study were carried out in accordance with the Australian Code of Practice for the Care and Use of Animals for Scientific Purposes 2004, the NSW Animal Research Act 1985, and the NSW Animal Research Regulation 2005.

## RESULTS

### Feed particle size distribution and pellet durability

**Figure 1:**
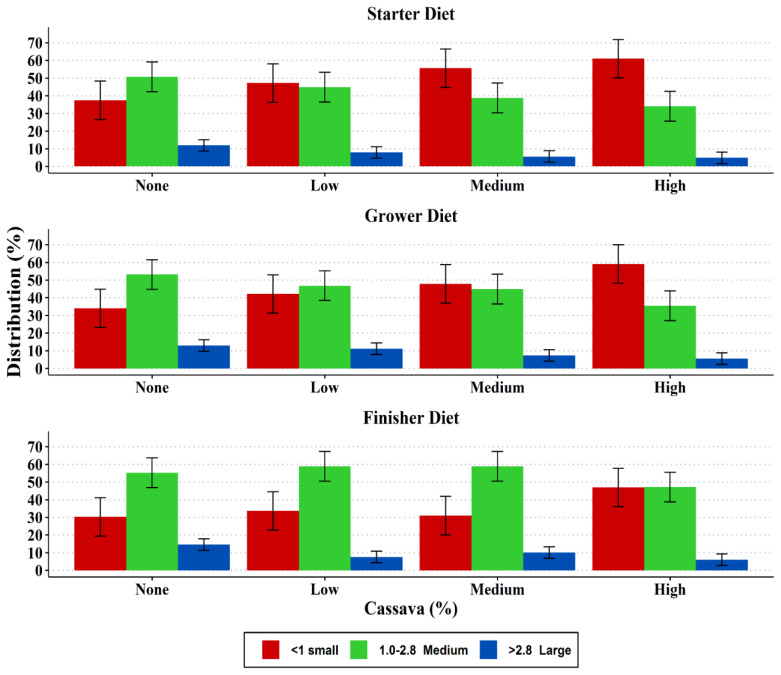
Particle size distribution of the experimental diets (T1–T4).

**Figure 2:**
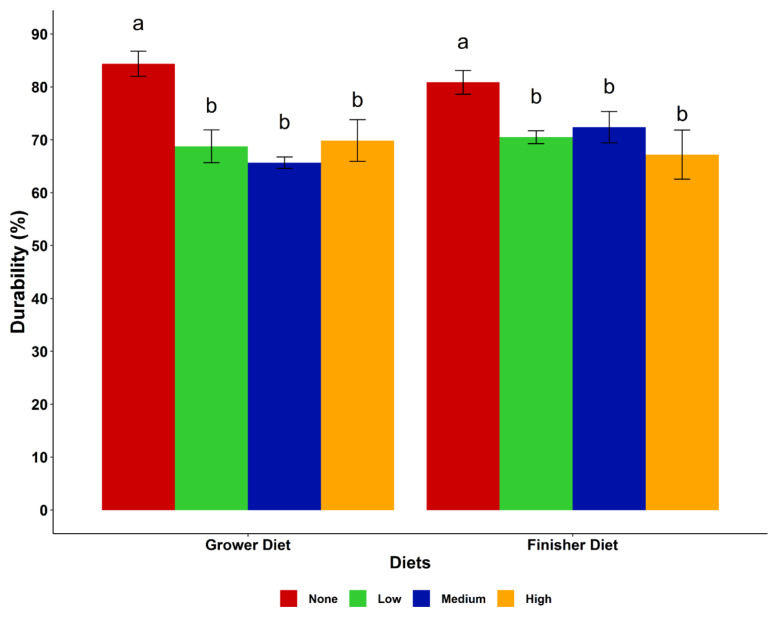
Effect of cassava levels on pellet durability of the dietary treatments. ^a,b^ Means with different superscripts within the same diet are significantly different (p<0.05). Cassava levels (none = 0%, low = 25%, medium = 50%, high = 75%).

The particle size distribution and pellet durability of the diets are presented in Figure 1 and 2, respectively. The particle size distribution in the starter, grower and finisher diets showed an increasing trend of small particles (<one mm) with increase in cassava level in the diet, while medium size particles (1.0 to 2.8 mm) decreased in the same direction. However, the medium particle sizes of the finisher diets were very similar in the none, low and medium cassava diets, but the diets with a high level of cassava had a lower amount. Larger sized particles (>2.8 mm) were fewer in all diets (<10%) with a decreasing trend as the cassava level increased. Including cassava in the diets decreased (p<0.05) pellet durability regardless of the level of cassava inclusion. The highest durability index (PDI) was observed in the control grower and finisher diets.

### Feed consumption and growth

Cassava inclusion increased (p<0.05) FI, with the highest value observed at 50% (medium) cassava for 1 to 24 d and 1 to 35 d of age (Table 4). However, the FI for 1 to 10 d was not affected (p>0.05) by cassava level. There was no effect (p<0.05) of enzyme supplementation on the FI of birds. The BWG linearly declined (p<0.05) with increase in cassava level. However, enzyme supplementation increased (p<0.05) the BWG of the birds. Increasing cassava level led to an increased FCR (p<0.05), which was further reduced (p<0.05) by enzyme addition. There was no noticeable interaction effect between the cassava level and enzyme supplementation on the FI, BWG, and FCR.

### Meat parts yield

The effects of cassava level and enzyme supplementation on dressing percentage (DP) and meat parts of broiler chickens are shown in Table 5. Absolute weights of breast, thighs and drumsticks were highest (p<0.001) at none and low levels of cassava, while at higher cassava levels the parts weight decreased (p<0.001). The absolute weights of all measured body parts increased (p<0.001) when enzymes were supplemented. The DP and relative weights of drumsticks declined (p<0.001) with increasing levels of cassava in the diets. The DP and relative weights of breast, thighs and drumsticks increased (p< 0.001) with enzyme supplementation. There was no interaction effect (p>0.05) between cassava level and enzyme supplementation on DP, nor with absolute and relative weights of the measured body parts.

### Bone morphology and mineral composition

Increasing level of cassava in the diets decreased (p<0.05) the ash content, weight, length, diameter and bone breaking strength of the tibia bone (Table 6). Supplementing diets with the enzymes increased (p<0.05) the ash content, weight and length of the tibia bone, with a slight increase in diameter and breaking strength. There was a positive interaction (p<0.01) between cassava level and enzyme supplementation on the diameter of the bone.

The tibia bone content of Ca, K, and Zn increased (p<0.001) with increasing level of cassava, while P and Mg content decreased (p<0.001) as the level of cassava in the diet increased (Table 7). The content of S was not affected (p>0.05) by cassava inclusion. Addition of Ronozyme A+VP increased the concentrations of all minerals in the tibia bone. There was no interaction (p>0.05) between treatments.

### Energy retention

The effect of cassava level and enzyme supplementation on body fat, protein content and energy retention of broiler chickens is presented in Table 8. Increasing the cassava level decreased (p<0.001) the body fat and energy retained as fat, while body protein and energy retained as protein was not affected (p>0.05) by cassava level. However, enzyme addition increased protein content and energy retention as protein. No interaction effect (p>0.05) was observed between treatments.

## DISCUSSION

### Feed particle size distribution and pellet durability

Particle size is an important characteristic of feed as it affects voluntary FI and the development of the gastrointestinal tract in avian species [18]. Therefore, a broiler diet with a balanced distribution of particles (small, medium, and large) is vital for performance enhancement. In the current study there was an increasing trend of small particles (<1 mm) and decreased large particles (>2.8 mm) as cassava levels in the diets increased. The reason for the high proportions of small-and medium-sized particle as well as fewer larger-sized particles in the diets in this study could be due to the processing of the cassava used in this study, which could have been broken down to predominantly fine and small particles during milling and feed mixing. Feeding diets with high proportions of small to medium feed particle sizes leads to greater exposure to digestive enzymes and presumably less energy needed for mechanical digestion [19]. Studies have shown that diets with more medium-to large-sized particles increase FI and utilization by influencing the development and the action of the gizzard, and hence improve performance of birds [14].

Pellet durability indicates the ability of the pellets to remain intact, with a low level of fines produced during handling, transportation and feeding [20]. In this study, pellet durability decreased with cassava inclusion, while the highest PDI was observed in the control grower and finisher diets. The high fibre and starch contents of maize compared to cassava might be the main reason behind this result. Furthermore, the difference in pellet durability in this study could be attributed to the quality of the feed ingredients used and moisture content of feeds. Literature supporting the effect of cassava on pellet durability is sparse. However, feeding birds pelleted diets with low PDI results in an accumulation of fine particles in the feed which leads to poor bird performance [12].

### Feed consumption and growth performance

The results of the current study have revealed that FI increased when birds were supplied with diets containing cassava between hatch and 24 d and over the entire production cycle. However, enzyme supplementation did not affect the FI. The digestibility of cassava starch has been found to be higher than that of maize [21], and the ratio of amylose to amylopectin in cassava (0.2) is less than the maize (0.38), as reported by Fallahi et al [22]. Other studies [9,23], have also showed lower amylose content in cassava root (17% to 19%) compared to that of maize (20% to 30%). These facts imply that cassava starch would be more highly digested than maize starch, thus the cassava-containing diets could have less retention time in the gut and thus increase the FI of the birds. Also, the cassava used in this experiment was in form of grits, and processing to grits may have improved the nutritive value although this was not directly assessed.

There was a reduction in the BWG of birds as the levels of cassava in the diets increased; however, enzyme supplementation resulted in heavier birds. In spite of the starch quality of cassava, most studies have reported a poorer performance with diets containing cassava compared to those with maize. Maize remains the premier energy source for the poultry industry but there is scope for use of cassava as has been shown in this and other studies. A detailed economic study was not conducted in this study but in cassava-producing areas, diets containing cassava would be cheaper than those containing maize. Inclusion of the test enzymes in this study could have increased digestion by hydrolysing the cell wall component and deactivating ANFs in cassava and other plant ingredients. Therefore, diets supplemented with microbial enzymes could release more nutrients, increase absorption and improve the performance of birds [24]. The results of the current study are in line with the work of Broch et al [8] who observed a linear reduction in BWG of the broiler birds when the dry residue of cassava level was increased in the maize–soybean-based diets.

The increased FCR in broiler birds fed medium and high levels cassava diets in this study is directly related to the high FI and low BWG. Previous findings [25] reported a poor FCR when higher levels of cassava were included in the diet. The low cassava-containing diets (<50%) in this study had lower FCR, but FCR was high with the diets containing the higher levels of cassava inclusion which is in line with previous findings by Rafiu et al [26], who found that FCR increased as cassava increases in the diets. This loss in efficiency could be directly related to the reduction in BWG. The FCR in this study was further improved by microbial enzyme supplementation.

### Meat yield

In this study, higher levels of cassava decreased the DP, absolute weight of breast, thighs and drumsticks, and relative weight of thighs. However, addition of microbial enzymes increased all measured carcass parameters. The decreased DP and weight of meat parts could be due to the depressed broiler performance as a result of increasing cassava in the diets since there is a direct relationship between growth and development of meat parts. This is supported by previous research [26,27] which all reported poor development of body components in birds consuming diets with high levels of cassava. The authors related the low meat yield to the deficiency of essential amino acids in cassava, especially lysine and methionine, in comparison to maize. However, some other studies [24] did not observe any significant difference in meat yield when different levels of cassava replaced maize in broiler diets. The variation between the results from different studies could be due to different varieties of cassava and enzymes used, methods of processing and the experimental environment.

### Bone quality

Tibia bone ash content, weight, length, width and breaking strength decreased with an increase in cassava level in the diets but were improved with microbial enzyme supplementation. The poor growth of birds fed cassava-containing diets resulted in smaller birds overall, which might then have contributed to smaller and weaker tibia bones as well as the imbalance of important minerals for bone quality such as Ca, P, and Mg. The concentrations of Ca, K, and Zn in tibia bone increased with an increasing cassava level in the current study, while P and Mg contents decreased as the level of cassava in the diet increased. This is a reflection of the concentration of these minerals in cassava and maize, where majority of mineral contents are higher in cassava than in maize. Previous study [28] found higher Ca, P, K, and lower Mg content in cassava than in maze. In this study, there was an increase in the contents of all minerals measured when diets were supplemented with the test enzymes. Addition of enzymes could have enhanced nutrient digestibility, including bone mineralization. There is limited published work on the effect of cassava on bone quality. However, in a study by Ologhobo et al [29]. was observed no effect of cassava on bone physical characteristics and mineral content.

### Energy retention

Body fat and energy retained as fat decreased as cassava level increased in the dietary treatments in this study. On the other hand, addition of microbial enzyme increased the protein content and energy retained as protein. Diets with high levels of cassava possibly had more fibre content which could lower the digestibility, availability and absorbability of the energy, which might affect its utilization and reduce the body energy content and retention. In this study, the addition of enzymes was able to restore some of these nutrients and improve their retention. Very little research has been undertaken in the area of nutrient utilization by broilers on cassava-based diets. However, the results of the current study are partially in agreement with previous findings [26] which reported high fat and protein retention in a group of broiler chickens fed maize-based diets compared to those on cassava-based diets. The authors also noticed an increase in fat and protein retention when a cocktail of enzymes containing carbohydrase, protease and phytase was supplemented.

## CONCLUSION

The findings of the present study indicated that cassava can replace maize by up to 50% without compromising growth performance, but with possible negative effects when cassava levels increased above 50%. The utilization of such diets can be further improved using appropriate microbial enzyme supplements, including α-amylase and β-glucanase, and enzymes that target the cell walls of feed ingredients of plant origin. Future studies need to focus on the economic implications of replacing maize with cassava in different combinations. Cassava can be more beneficial to broiler chickens when properly processed and supplemented with microbial enzymes. The results of this study will be useful in introducing a new energy source (cassava) to the poultry industry in Tanzania and other countries which produce cassava on a commercial basis.

## Figures and Tables

**Table 1:** Feed ingredient composition (g/kg) of the test diets1)

Cassava level	Starter				Grower				Finisher			
		
	0%	25%	50%	75%	0%	25%	50%	75%	0%	25%	50%	75%
Maize	623.2	455.8	300.9	148.4	597.5	440.2	289.1	142.1	646.6	476.8	309.7	134.8
Cassava	0.0	152.0	301.0	445.7	0.0	146.8	289.1	426.6	0.0	159.0	309.7	466.1
Soybean meal	207.0	233.8	235.8	241.8	225.4	240.1	244.2	239.6	186.0	192.7	223.6	224.4
Cottonseed meal	92.8	81.2	89.6	88.5	78.4	76.9	79.2	90.9	68.6	70.4	63.9	75.4
Fish meal	23.9	22.0	23.1	22.8	29.7	30.3	32.4	34.6	15.7	19.3	15.0	15.1
Canola oil	0.5	0.3	2.6	4.3	17.7	17.3	17.0	18.6	36.3	37.6	35.7	40.2
Dicalcium phosphate	13.6	10.0	6.0	2.7	14.7	14.4	14.2	14.0	12.2	12.0	11.6	11.7
Limestone	9.5	13.6	11.3	12.1	8.8	8.6	8.5	8.3	6.9	6.2	5.8	4.9
Mineral premix2)	8.6	9.9	8.3	8.2	8.8	8.6	8.5	8.3	8.8	8.7	8.5	8.4
Vitamin premix3)	6.7	8.4	7.8	6.4	6.8	6.7	6.6	6.5	8.0	6.7	6.6	6.6
L-lysine HCl	5.1	4.3	4.8	4.8	3.9	2.1	3.1	3.0	3.5	3.4	2.9	2.3
L-threonine	2.9	2.8	2.8	8.1	2.0	1.9	1.9	2.0	1.5	1.5	1.4	1.8
DL-methionine	1.8	1.9	2.0	2.2	1.6	1.7	2.1	2.0	1.6	1.6	1.9	1.9
Sodium bicarbonate	2.7	2.4	2.7	2.8	2.1	1.9	1.9	1.6	2.1	2.0	1.8	1.5
Salt	0.8	1.0	0.8	0.7	1.5	1.6	1.6	1.5	1.4	1.5	1.6	1.7
Choline chloride 70%	0.8	0.6	0.4	0.3	0.8	0.6	0.4	0.2	0.8	0.6	0.3	3.0
HiPhos.	0.1	0.1	0.1	0.1	0.1	0.1	0.1	0.1	0.1	0.1	0.1	0.1

1) Diets were made by replacing maize with cassava levels (none, 0%; low, 25%; medium, 50%; high, 75%); half of each diet was added with enzymes = (Ronozyme A + Ronozyme VP at 500 g/tonne of diets); Ronozyme A contains α-amylase and β-glucanase, while Ronozyme VP contains cellulase and hemicellulases and pectinases.

2) Mineral premix (DM basis) contains: P, 0.1 g/kg; K, 0.08 g/kg; Cl, 0.27 g/kg; S, 3.05 g/kg; Se, 0.80 g/kg; Zn, 266 g/kg; Fe, 106 g/kg; Cu, 42.67 g/kg; Mn, 240 g/kg.

3) Vitamin premix (95.8%) contains vitamin A, 24,000,000 IU; vitamin E, 150,000 IU; vitamin K, 6 g/kg; Thiamin, 6 g/kg; Riboflavin, 16 g/kg; Niacin, 110,000 g/kg; Pantothenic acid, 26 g/kg; Pyridoxine, 10 g/kg; Folacin, 4 g/kg; Biotin, 0.5 g/kg. HiPhos (Base enzyme), phytase.

**Table 2:** Nutrient composition of dietary treatments

Items	Starter	Grower	Finisher
ME (kcal/kg)	3,000	3,100	3,200
ME (MJ/kg)	12.6	13.0	13.4
CP (g/kg)	230	215	196
Crude fat (g/kg)	27	55	60
Crude fibre (g/kg)	41	38	33
Arginine (g/kg)	15	14	13
Lysine (g/kg)	13	12	10
Methionine (g/kg)	5	5	4
Methionine+cystine (g/kg)	10	9	8
Tryptophan (g/kg)	2	2	2
Leucine (g/kg)	14	13	11
Isoleucine (g/kg)	8	8	7
Threonine (g/kg)	9	8	7
Valine (g/kg)	9	9	8
Calcium (g/kg)	10	9	8
Phos. Avail (g/kg)	5	5	4
Sodium (g/kg)	2	2	2
Chloride (g/kg)	2	2	2
Choline (mg/kg)	1,700	1,600	1,500

The diets in the three phases had similar nutritional composition.

ME, metabolizable energy; CP, crude protein.

**Table 3:** Nutrient composition (g/kg) of cassava grit used in feed formulation

Items	
Macro minerals	
Nitrogen	3.12
Phosphorus	1.38
Potassium	12.77
Sulphur	0.28
Calcium	1.20
Magnesium	0.94
Sodium	0.17
Micro minerals	
Copper	0.003
Zinc	0.008
Manganese	0.012
Iron	0.033
Proximate components	
Crude protein	20
Oil content	5
Crude fibre	40
Total sugars	22
Energy (MJ/kg)	17
Total starch	870
Amino acids	
Arginine	0.7
Threonine	0.5
Lysine	0.8
Methionine	0.1
Valine	0.6
Isoleucine	0.5
Leucine	0.8
Phenylalanine	0.5

**Table 4:** Effect of cassava level and enzyme supplementation on feed intake (FI), body weight gain (BWG), and feed conversion ratio (FCR)

Items	Enzymes1)	FI (g)			BWG (g)			FCR		
		
		1–10 d	1–24 d	1–35 d	1–10 d	1–24 d	1–35 d	1–10 d	1–24 d	1–35 d
Cassava level2)										
None	None	241.7	1,527.5	3,251.1	234.7	1,301.5	2,329.2	0.99	1.18	1.41
	Plus	269.7	1,588.2	3,351.5	253.0	1,407.1	2,464.3	1.03	1.13	1.37
Low	None	269.8	1,637.1	3,451.7	235.0	1,173.4	2,299.1	1.10	1.41	1.52
	Plus	253.2	1,573.8	3,341.4	246.9	1,227.4	2,410.1	0.99	1.30	1.40
Medium	None	252.0	1,630.5	3,411.0	220.0	1,189.0	2,041.3	1.12	1.39	1.69
	Plus	260.0	1,707.5	3,510.9	232.2	1,230.5	2,314.9	1.08	1.39	1.53
High	None	283.8	1,637.2	3,409.5	219.5	1,117.1	1,987.7	1.24	1.47	1.72
	Plus	273.9	1,664.6	3,453.4	233.9	1,225.5	2,070.3	1.10	1.36	1.67
SEM		3.67	12.50	21.60	2.91	18.60	35.0	0.02	0.02	0.03
Main effects										
Cassava										
None		255.7	1,557.8^b^	3,301.3^b^	243.8^a^	1,354.3^a^	2,396.8^a^	1.01^b^	1.16^b^	1.39^c^
Low		261.5	1,605.4^ab^	3,396.5^ab^	241.0^ab^	1,200.4^b^	2,354.6^ab^	1.05^ab^	1.35^a^	1.46^bc^
Medium		256.0	1,669.0^a^	3,460.9^a^	226.1^b^	1,209.8^b^	2,178.1^bc^	1.10^ab^	1.39^a^	1.61^ab^
High		278.8	1,650.9^a^	3,431.5^ab^	226.7^b^	1,171.3^b^	2,028.94^c^	1.17^a^	1.41^a^	1.69^a^
Enzyme										
	None	261.8	1,608.1	3,380.8	227.3^b^	1,195.3^b^	2,164.3^b^	1.11	1.36	1.58^a^
	Plus	264.2	1,633.5	3,414.3	241.5^a^	1,272.6^a^	2,314.9^a^	1.05	1.30	1.49^b^
Source of variation										
Cassava		0.071	0.003	0.044	0.037	0.001	0.001	0.022	0.001	0.001
Enzyme		0.732	0.241	0.412	0.011	0.017	0.008	0.079	0.060	0.049
Cassava×enzyme		0.112	0.109	0.224	0.973	0.817	0.606	0.356	0.599	0.726

FI, feed intake; WG, weight gain; FCR, feed conversion ratio; SEM, standard error of the means, each value represents the mean of 6 replicates (10 birds per replicate).

1) Enzymes = Ronozyme A + Ronozyme VP at 500 g/tonne of diets.

2) Cassava levels: none, 0%; low, 25%; medium, 50%; high, 75%.

a–c Means with different superscripts within the columns are significantly different (p<0.05).

**Table 5:** Effect of cassava level and enzyme supplementation on the meat yield

Items	Enzyme1)	Absolute weight (g)			DP %	Relative weight (g/kg)		
	
		Breast	Thigh	Drumsticks		Breast	Thigh	Drumsticks
Cassava level2)								
None	None	331.3	199.6	156.8	71.9	171.7	103.7	81.0
	Plus	427.2	229.6	204.7	74.0	209.7	112.6	100.5
Low	None	306.1	189.7	177.2	71.7	158.0	98.2	92.2
	Plus	412.5	224.6	210.2	72.0	215.9	117.3	109.9
Medium	None	281.5	163.6	156.6	70.4	170.7	99.0	95.2
	Plus	393.9	214.7	222.9	71.6	207.2	113.8	116.7
High	None	270.1	153.1	133.5	66.1	164.3	93.2	82.1
	Plus	337.4	188.2	164.8	70.6	194.9	109.1	95.5
SEM		9.87	4.56	5.38	0.41	3.77	1.62	2.26
Main effect								
Cassava								
None		379.2^a^	214.6^a^	180.8^a^	73.0^a^	190.7	108.2	90.7^b^
Low		359.3^a^	207.1^ab^	193.7^a^	71.8^a^	187.0	107.8	101.0^ab^
Medium		337.7^ab^	189.1^b^^c^	189.8^a^	71.0^a^	188.9	106.4	105.9^a^
High		303.7^b^	170.6^c^	149.1^b^	68.3^b^	179.6	101.2	88.8^b^
Enzyme								
	None	297.2^b^	176.5^b^	156.0^b^	70.0^b^	166.2^b^	98.5^b^	87.6^b^
	Plus	392.7^a^	214.3^a^	200.7^a^	72.0^a^	206.9^a^	113.2^a^	105.7^a^
Source of variation								
Cassava		0.001	0.001	0.001	0.001	0.347	0.166	0.001
Enzyme		0.001	0.001	0.001	0.001	0.001	0.001	0.001
Cassava×enzyme		0.560	0.575	0.285	0.076	0.186	0.519	0.836

DP, dressing percentage; EM, standard error of the means.

1) Enzymes = (Ronozyme A + Ronozyme VP at 500 g/tonne of diets).

2) Low, 25%; medium, 50%; high, 75%.

a,b Means with different superscripts within the columns are significantly different (p<0.05).

**Table 6:** Effect of cassava level and enzyme supplementation on tibia bone physical characteristics in broiler chickens

Items	Enzyme1)	Ash (%)	Bone measurements			

			WT (g)	L (mm)	D (mm)	Load (N)
Cassava level2)						
None	None	47.9	8.3	87.0	14.8	236.4
	Plus	48.1	9.9	91.3	15.9	286.1
Low	None	47.0	8.1	85.6	15.0	254.8
	Plus	47.6	10.6	88.6	14.8	231.3
Medium	None	46.1	8.7	84.1	14.2	212.4
	Plus	48.7	9.9	88.9	15.8	230.8
High	None	45.5	7.7	83.7	14.4	206.3
	Plus	46.5	9.1	86.2	14.6	202.5
SEM		0.27	0.17	0.44	0.12	5.88
None	None	47.9	8.3	87.0	14.8	236.4
	Plus	48.1	9.9	91.3	15.9	286.1
Low	None	47.0	8.1	85.6	15.0	254.8
	Plus	47.6	10.6	88.6	14.8	231.3
SEM		0.27	0.17	0.44	0.12	5.88
Main effect						
Cassava level						
None		48.0^a^	9.1^ab^	89.2^a^	15.4^a^	261.3^a^
Low		47.3^ab^	9.3^a^	87.1^ab^	14.9^ab^	243.0^ab^
Medium		47.4^ab^	9.3^a^	86.5^b^	15.0^ab^	221.6^bc^
High		46.0^b^	8.3^b^	85.0^b^	14.5^b^	204.4^c^
Enzyme		48.0^a^	9.1^ab^	89.2^a^	15.4^a^	261.3^a^
	None	46.6^b^	8.2^b^	85.1^b^	14.6	227.5
	Plus	47.7^a^	9.9^a^	88.8^a^	15.3	237.7
Source of variation						
Cassava level		0.044	0.009	0.001	0.023	0.001
Enzyme		0.034	0.001	0.001	0.001	0.302
Cassava level×enzyme		0.377	0.124	0.454	0.013	0.068

WT, weight; L, length; D, diameter; Load, load, or breaking strength measured in Newtons; SEM, standard error of the means, each value represents the mean of 6 replicates (10 birds per replicate).

1) Enzymes = (Ronozyme A + Ronozyme VP at 500 g/tonne of diets).

2) Cassava levels: none, 0%; low, 25%; medium, 50%; high, 75%.

a–c Means with different superscripts within the columns are significantly different (p<0.05).

**Table 7:** Effect of cassava level and enzyme supplementation on tibia bone mineral composition (mg/kg) of broilers

Items	Enzyme1)	Ca	P	K	Mg	S	Zn
Cassava level2)							
None	None	396.0	182.4	7.4	8.8	3.0	471.3
	Plus	399.7	186.1	7.7	8.9	3.7	536.2
Low	None	391.8	180.5	7.2	8.5	2.9	532.6
	Plus	401.1	184.2	7.7	8.6	3.8	624.4
Medium	None	403.5	181.1	7.5	8.3	3.0	537.7
	Plus	406.9	182.6	7.7	8.4	3.7	656.8
High	None	400.1	177.8	7.7	8.2	3.1	644.5
	Plus	410.8	181.6	8.2	8.5	3.6	717.5
SEM		1.06	0.46	0.05	0.04	0.07	13.50
Main effect							
Cassava level							
None		398.0^b^	184.3^a^	7.5^b^	8.8^a^	3.4	503.8^c^
Low		396.4^b^	182.4^a^	7.5^b^	8.6^b^	3.3	578.5^bc^
Medium		405.2^a^	181.9^ab^	7.6^b^	8.4^b^	3.4	597.2^b^
High		405.5^a^	179.7^b^	7.9^a^	8.3^b^	3.4	681.0^a^
Enzyme							
	None	397.8^b^	180.5^b^	7.4^b^	8.4^b^	3.0^b^	546.5^b^
	Plus	404.6^a^	183.7^a^	7.8^a^	8.6^a^	3.7^a^	633.7^a^
Source of variation							
Cassava		0.001	0.001	0.001	0.001	0.994	0.001
Enzyme		0.001	0.001	0.001	0.018	0.001	0.001
Cassava level×enzyme		0.196	0.571	0.248	0.769	0.755	0.641

SEM, standard error of the means, each value represents the mean of 6 replicates (10 birds per replicate).

1) Enzymes = Ronozyme A + Ronozyme VP at 500 g/tonne of diets.

2) Cassava levels: none, 0%; low, 25%; medium, 50%; high, 75%.

a–c Means with different superscripts within the columns are significantly different (p<0.05).

**Table 8:** Body fat and protein content (g/BWT), and energy retention (kJ/d) of broiler chickens fed diets with different cassava levels supplemented with microbial enzymes

Items	Enzyme1)	Fat (g of Bwt)	CP (g of Bwt)	ER.Fat (kJ/d)	ER.Protein (kJ/d)
Cassava level2)					
None	None	121.4	268.0	210.8	287.5
	Plus	140.1	283.3	243.3	303.9
Low	None	116.6	241.9	202.5	259.5
	Plus	112.7	255.3	195.7	273.9
Medium	None	111.3	240.8	193.3	258.3
	Plus	104.3	258.6	181.1	277.5
High	None	88.6	245.8	153.9	263.7
	Plus	104.0	267.7	180.6	287.2
SEM		2.87	4.30	4.99	4.61
Main effect					
Cassava					
None		130.8^a^	275.6	227.1^a^	295.7
Low		114.7^ab^	248.6	199.1^ab^	266.7
Medium		107.8^bc^	249.7	187.2^bc^	267.9
High		96.3^c^	256.7	167.2^c^	275.4
Enzyme					
	None	109.5	249.1^b^	190.1	267.6^b^
	Plus	115.3	266.2^a^	202.2	285.6^a^
Source of variation					
Cassava		0.001	0.091	0.001	0.091
Enzyme		0.192	0.044	0.192	0.044
Cassava×enzyme		0.098	0.985	0.098	0.985

SEM, standard error of the means, each value represents the mean of 6 replicates (10 birds per replicate); Fat (gBwt), fat content of birds’ live body weight (g); CP (gBwt), protein content of birds’ live body weight.

1) Enzymes = (Ronozyme A + Ronozyme VP at 0.50 g/tonne of diets).

2) Cassava levels: none, 0%; low, 25%; medium, 50%; high, 75%.

a–c Means with different superscripts within the columns are significantly different (p<0.05).
